# SARS-CoV-2 infection in brown-headed spider monkeys (*Ateles fusciceps*) at a wildlife rescue center on the coast of Ecuador—South America

**DOI:** 10.1128/spectrum.02741-23

**Published:** 2024-02-16

**Authors:** Mateo Carvajal, Carolina Saenz, Nathalia Fuentes, Rommel Guevara, Erika Muñoz, Belen Prado-Vivar, Eduardo Diaz, Felipe Alfonso-Cortes, Josefina Coloma, Michelle Grunauer, Patricio Rojas-Silva, Paul A. Cardenas, Veronica Barragan

**Affiliations:** 1 Universidad San Francisco de Quito, Instituto de Microbiología, Quito, Ecuador; 2 Universidad San Francisco de Quito, Hospital de Fauna Silvestre TUERI-USFQ, Quito, Ecuador; 3 Proyecto Washu-Fundación Naturaleza y Arte, Quito, Ecuador; 4 Universidad San Francisco de Quito, Escuela de Medicina Veterinaria, Quito, Ecuador; 5 University of California, Berkeley, California, USA; 6 Universidad San Francisco de Quito, Escuela de Medicina, Quito, Ecuador; Emory University School of Medicine, Atlanta, Georgia, USA

**Keywords:** Ecuador, wildlife, SARS-CoV-2, *Ateles fusciceps*, Nanopore, omicron

## Abstract

Human populations can be affected in unpredictable ways by the emergence and spread of zoonotic diseases. The COVID-19 (coronavirus disease of 2019) pandemic was a reminder of how devastating these events can be if left unchecked. However, once they have spread globally, the impact of these diseases when entering non-exposed wildlife populations is unknown. The current study reports the infection of brown-headed spider monkeys (*Ateles fusciceps*) at a wildlife rescue center in Ecuador. Four monkeys were hospitalized, and all tested positive for SARS-CoV-2 (Severe acute respiratory syndrome coronavirus 2) by RT-qPCR (Quantitative Reverse Transcription PCR). Fecal samples (*n* = 12) from monkeys at the rescue center also tested positive; three zookeepers responsible for feeding and deworming the monkeys also tested positive, suggesting human-animal transmission. Whole genome sequencing identified most samples’ omicron clade 22B BA.5 lineage. These findings highlight the threat posed by an emerging zoonotic disease in wildlife species and the importance of preventing spillover and spillback events during epidemic or pandemic events.

**IMPORTANCE:**

Although COVID-19 (coronavirus disease of 2019) has been primarily contained in humans through widespread vaccination, the impact and incidence of SARS-CoV-2 (Severe acute respiratory syndrome coronavirus) and its transmission and epidemiology in wildlife may need to be addressed. In some natural environments, the proximity of animals to humans is difficult to control, creating perfect scenarios where susceptible wildlife can acquire the virus from humans. In these places, it is essential to understand how transmission can occur and to develop protocols to prevent infection. This study reports the infection of brown-headed spider monkeys with SARS-CoV-2, a red-listed monkey species, at a wildlife recovery center in Ecuador. This study reports the infection of brown-headed spider monkeys with SARS-CoV-2, indicating the potential for transmission between humans and wildlife primates and the importance of preventing such events in the future.

## INTRODUCTION

Zoonotic spillover is a significant concern for the protection of human health and the prevention of future pandemics. An estimated 60%–75% of all emerging human infectious diseases is considered zoonotic (1, 2), and several have impacted the human population over time. Some of the most prominent cases include HIV, which originated in non-human primates (3, 4); Ebolavirus that was transmitted to humans from various wildlife species such as bats, chimpanzees, gorillas, and monkeys (5, 6). SARS-CoV (Severe acute respiratory syndrome) and MERS-CoV (Middle East respiratory syndrome–related coronavirus) were transmitted to human populations from bats through palm civets and dromedary camels as intermediate hosts, respectively (7). The impact of the pandemic recently experienced demonstrates the devastating effects of a new zoonotic virus jumping from non-human animals to a naïve population of humans. SARS-CoV-2, the pathogen responsible for the COVID-19 (coronavirus disease of 2019) pandemic that shook the world in 2020 and has claimed millions of lives, is a vivid example of zoonotic spillover, according to the most reliable data (8). Despite mitigation efforts, the exponential increase in SARS-CoV-2 infections resulted in a significant global health burden with unprecedented consequences (9).

The possibility of anthroponosis or spillback events is another concern. As with SARS-CoV-2, human cases have been reported everywhere, raising the potential of the virus spreading to wildlife. To date (November 2023), the SARS-ANI VIS database (A Global Open Access Dataset of Reported SARS-CoV-2 Events in Animals) (10) has reported 900 SARS-CoV-2 events in 34 animal species from 39 countries around the world, with a fatality rate of 1.4% (https://github.com/amel-github/sars-ani/). Among these events, only a few (*n* = 190) were reported in animals living in natural reserves or wildlife. Cases of SARS-CoV-2 in farmed mink demonstrated for the first time that the virus could infect these mammals, allowing new mutations to emerge and then spread back to humans (11); other domestic or farm animals may have transmitted the infection directly or indirectly to wildlife (12). For instance, free-ranging white-tailed deer in the USA are highly susceptible to SARS-CoV-2, and recent evidence has shown spillback to humans (11), suggesting that this species can be considered a new natural reservoir of the virus. Moreover, white-tailed deer can maintain transmission in the wild. A new variant has even evolved in these animals (13), highlighting the importance of monitoring this pathogen’s presence in wildlife.

Reports of SARS-CoV-2 infection in wildlife are scarce. Cases of SARS-CoV-2 infection have been reported in gorillas and squirrel monkeys in USA zoos. All the infected gorillas recovered well, but the squirrel monkey unfortunately died. The exact role of SARS-CoV-2 in the animal’s clinical presentation and demise is unclear (14–16). However, because non-human primates are primary models for SARS-CoV-2 in pathogenesis and vaccine development, the pathology of this infection is well known. The current study reports the identification of SARS-CoV-2 in brown-headed spider monkeys (*Ateles fusciceps*) living at a wildlife rescue center on the coast of Ecuador and a likely spillback event from humans to primates.

Understanding the routes of transmission of SARS-CoV-2 to these animal populations may help prevent transmission and motivate site-specific protocols that contribute to the prevention of infection. This study reports the identification of SARS-CoV-2 in brown-headed spider monkeys (*Ateles fusciceps*) living in a wildlife sanctuary on the coast of Ecuador describes possible transmission routes and a likely human-to-primate transmission event.

## MATERIALS AND METHODS

### Study site and background

RT-qPCR detected SARS-CoV-2 in *Ateles* monkeys at a wildlife rescue center located approximately 100 km from Guayaquil, near the Ecuadorian Pacific coast in Guayas. The rescue center has a unique infrastructure that includes a series of artificial islands with enclosures to keep and rehabilitate brown-headed spider monkeys (*Ateles fusciceps*) rescued from the illegal wildlife trade.

The site has two main areas (Fig. S1): area 1 contains a kitchen room for food preparation, and a few meters away, there is a cage enclosure (enclosure 1) with one brown-headed spider monkey. This monkey is not in direct contact with any other animals. Area 2 is 130 meters from Area 1 and consists of six small artificial islands with vegetation and trees (20 × 40 meters). A water stream interconnects the islands. Island 1 has two brown-headed spider monkeys, Island 2 has one, Island 3 is empty, and Islands 4, 5, and 6 have three, six, and five monkeys, respectively. There are three more cage enclosures (enclosures 2, 3, and 4) near the islands, each with one or two monkeys. Squirrel monkeys (*Saimiri cassiquiarensis*) frequently visit Islands 2 and 3 and enclosures 2, 3, and 4. These monkeys roam freely in the reserve in search of food. No squirrel monkeys have been recorded on islands 4, 5, and 6.

### Sample collection

Between 15 and 17 August 2022, 22 monkeys were dewormed using baits (a mixture of guayaba jam, baby cereal, and 2, 8, or 3.5 mL of nitazoxanide 100 mg/5 mL). On 22 August, three of these animals presented lethargy, anorexia, diarrhea, and fever, initially suspecting that it was due to the deworming process. Nevertheless, due to previous experience of the veterinarians, they suspected SARS-CoV-2.

**Fig 1:**
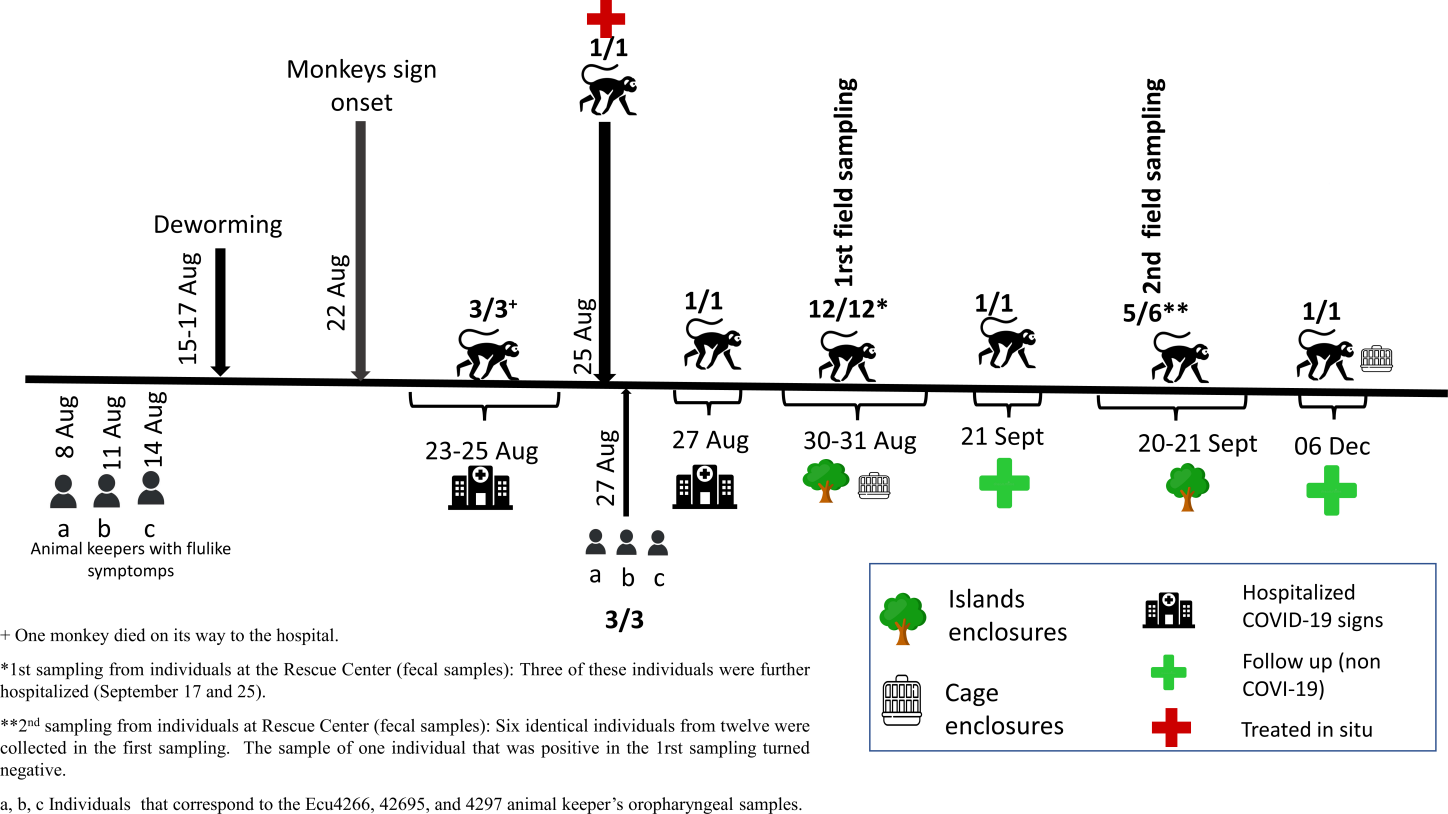
Chronology of sample collection. Numbers represent positive samples/total samples. Sample details are shown in Table 1. The hospital icon indicates hospitalized monkeys, and the tree icon indicates monkeys that are kept in artificial island enclosures. Cage icon indicates monkeys that were confined in cage enclosures at the rescue center. Sampling dates indicate when fecal samples were collected from monkeys in the rescue center. Human icons represent dates when animal keepers show flu-like symptoms and when their samples were collected for sequencing.

Between 23 August and 6 December 2022, nasal/fecal/tissue samples were collected from 15 adults and one juvenile brown-headed spider monkey living in the wildlife rescue center (Table S1). For the other six monkeys, taking samples was impossible due to logistic difficulties. The chronology of the sample collection is shown in Fig. 1. Briefly, on 23 August, the first monkey was transferred to Hospital de Fauna Silvestre TUERI-USFQ; this animal presented lethargy and was anorexic (Atl.16). It died on its way to the hospital; from this specimen, the first sample to conduct SARS-CoV-2 testing (lung tissue sample) was taken. Fecal and nasal swab samples were collected from other animals presenting to TUERI-USFQ (Atl.13,14,15) with signs similar to Atl.16 plus diarrhea and fever.

**TABLE 1:** RT-qPCR positivity, sampling dates, and SARS-CoV-2 clade and lineage assignment among monkeys and animal keepers

Sample	Type	Sampling date	RT-qPCR Cq^*b*^ (N1, N2 genes)	Cq-positive controls	Cq-negative controls	Clade	Lineage
Monkeys							
Atl.1^*a*^	Swab	17–09-2022	33.29, 31.47	22.48,20.99	NA^*c*^	Unassigned	Unassigned
	Feces 1	30–08-2022	36.49, 36.47	22.54,21.26	44.53, NA	Unassigned	Unassigned
	Feces 2	21–09-2022	35.07, 34.81	22.48,20.99	NA	NS^*d*^	NS
Atl.2	Feces 1	30–08-2022	38.27, 37.26	22.54,21.26	44.53, NA	NS	NS
Atl.3	Swab	17–09-2022	35.96, 33.96	22.48,20.99	NA	22B	Unassigned
	Feces 1	30/8/202	35.25, 33.13	22.54,21.26	44.53, NA	22B	B.1.1.529 (OQ630011.1)^*e*^
	Feces 2	21–09-2022	Negative	22.48,20.99	NA	NS	NS
Atl.4	Feces 1	30–08-2022	38, 38.8	22.54,21.26	44.53, NA	NS	NS
Atl.5	Feces 1	30–08-2022	38.67, 39.03	22.54,21.26	44.53, NA	NS	NS
	Feces 2	21–09-2022	34.25, 33.16	22.48,20.99	NA	NS	NS
Atl.6	Feces 1	30–08-2022	37.51, 38.87	22.54,21.26	44.53, NA	NS	NS
	Feces 2	21–09-2022	35.46, 34.72	22.48,20.99	NA	NS	NS
Atl.7	Swab	21/9/2022**	36.63, 35.1	22.48,20.99	NA	22B	Unassigned
	Feces 1	30–08-2022	34.98, 35.95	22.54,21.26	44.53, NA	Unassigned	Unassigned
Atl.8	Feces 1	31–08-2022	38.27, 39.30	22.54,21.26	44.53, NA	NS	NS
Atl.9	Feces 1	31–08-2022	35.39, 34.87	22.54,21.26	44.53, NA	22B	B.1.1.529 (PP265987)^*e*^
Atl.10	Feces 1	31–08-2022	38.14, 37.65	22.54,21.26	44.53, NA	NS	NS
	Feces 2	6/12/2022**	Negative	22.88,22.64	NA	NS	NS
Atl.11	Feces 1	31–08-2022	35.05, 34.34	22.54,21.26	44.53, NA	22B	B.1.1.529 (OQ630004)^*e*^
	Feces 2	20–09-2022	35.84, 34.65	22.48,20.99	NA	NS	NS
Atl.12	Feces 1	31–08-2022	36.49, 36.79	22.54,21.26	44.53, NA	NS	NS
	Feces 2	21–09-2022	36.74, 35.42	22.48,20.99	NA	NS	NS
Atl.13	Swab	25–08-2022	35.79, 32.99	25.77,25.33	NA	22B	BA.5.2 (OQ630005)^*e*^
	Feces ***	30–08-2022	34.96, 33.63	22.54,21.26	44.53, NA	22B	Unassigned
Atl.14	Swab	29–08-2022	32.15, 30.98	25.77,25.33	NA	22B	B.1.1.529 (OQ630007)^*e*^
	Feces ***	20–09-2022	34.79, 34.81	22.48,20.99	NA	22B	B.1.1.529 (OQ630006)^*e*^
Atl.15	Swab	24–08-2022	32.99, 33.79	25.77,25.33	NA	22B	B.1.1.529 (OQ630008)^*e*^
Atl.16	Tissue	24–08-2022	36.55, 34.76	25.77,25.33	NA	22B	B.1.1.529 (2459402485)^*e*^
Monkey keepers							
Ecu4266	Swab	27–08-2022	33.37,32.7	23.59,22.47	44.53, NA	22B	Unassigned
Ecu4295	Swab	27–08-2022	34.86,34.44	23.59,22.47	44.53, NA	22B	B.1.1.529 (OQ630012)^*e*^
Ecu4297	Swab	27–08-2022	31.47,30.67	23.59,22.47	44.53, NA	22B	B.1.1.529 (OQ630012.1)^*e*^

^*a*^ Atl: Ateles fusciceps.

^*b*^ RT-qPCR Cq: Quantitative Reverse Transcription PCR quantification cycle.

^*c*^ NA: not applicable.

^*d*^ NS: no sample.

^*e*^ GenBanK.

Additionally, fecal samples were collected on two different dates (*n* = 12 on 30–31 August and *n* = 6 resampled on 20–21 September), from apparently healthy individuals living in the rescue center (see Table S1 for sample details). On 25 August, one of the monkeys (Atl.3) showed signs of lethargy and anorexia and was treated on-site. Finally, nasal swab samples were taken from Atl.1 and 3. These animals came to the hospital for a follow-up of previous hospitalization (non-COVID-19 related). All samples were collected for diagnostic purposes by veterinarians using standard protocols from the Hospital de Fauna Silvestre TUERI-USFQ, under the Ministerio del Ambiente, Agua y Transición Ecológica permit MAATE-ARSFC-2022-2737.

To ensure the preservation of viral genomic material, all samples were placed in RNA/DNA Shield (Zymo, USA) immediately after collection. To guarantee the collection of fresh fecal samples, animals living on the artificial islands were followed visually until defecation occurred. KN95 disposable masks and gloves (a pair per sample) were always worn to avoid cross-contamination.

Three of four animal keepers showed flu-like symptoms between 8 and 14 August 2022. Those people were not involved in the process of collecting the animal samples. On 27 August, we collected oropharyngeal swab samples from animal keepers under the Bioethics Committee of Universidad San Francisco de Quito (CEISH No. P2020-022IN) and by the Ministerio de Salud del Ecuador (MSP-CGDES-2020-0121-O). Swabs were stored in RNA/DNA Shield (Zymo, USA) and transported to the Institute of Microbiology at Universidad San Francisco de Quito for further processing.

### SARS-CoV-2 detection and sequencing

Viral RNA from samples was extracted using the Zymo Quick-RNA Viral Kit (Zymo, USA) and Qiagen RNeasy Mini Kit (QIAGEN, Germany) following the manufacturer’s modifications for feces and tissues. Briefly, 20 µL of proteinase K and 180 µL of PK digestion buffer were added to 3–5 mg of stool or tissue and incubated at 55°C overnight. Then, the digestion product was processed according to kit inserts. Molecular virus detection was performed using the Luna SARS-CoV-2 RT-qPCR Multiplex Assay Kit (New England Biolabs-NEB, USA). All samples were tested in duplicate; we used negative and positive controls in each run. Ct values higher than 40 were considered negatives.

Whole genome sequencing was performed following the Artic-nCoV-2019 protocol v3 (17). For PCR multiplex amplification of the SARS-CoV-2 genome, the NEBNext VarSkip Short v2 primers (New England Biolabs-NEB, USA) and the Midnight amplicon panel (18) were used. The sequencing library was prepared using the Oxford Nanopore Native Barcoding Expansion 96 (EXP-NBD196) with Ligation Sequencing Kit LSK-109 and loaded into an R9.4.1 flow cell in a GridION Mk1. The sequencing run was programmed with the MinKNOW software v22.08.6, and real-time demultiplexing and adapter trimming were activated with the high-accuracy mode for basecalling.

The Fastq reads obtained from the same sample taken on the same date but generated with different extraction kits and primer panels were combined. EPI2ME Labs wf-artic workflow v0.3.18 (https://github.com/epi2me-labs/wf-arti) was used to produce consensus sequences and alignments which were visualized on Tablet v1.21.02.08 (19). Nextclade v2.5.0 (20) web servers were used for clade assignment. Alternatively, to improve lineage assignment, the nucleotide sequences of the S gene were extracted with AliView (21) and Hedgehog v1.6 (22) was used for Pango lineage assignment, only sequences with more than 50% of coverage were used. For the sequences that were not assigned due to the high number of missing data, the amino acid substitutions were reviewed and compared with the defining mutations of the omicron variant.

## RESULTS

Four brown-headed spider monkeys (Atl.13, 14, 15, and 16) were admitted to Hospital de Fauna Silvestre TUERI-USFQ between 23 and 27 August 2022. The symptoms that some of them presented were lethargy, anorexia, diarrhea, and fever. All four animals tested positive for SARS-CoV-2 (Fig. 1 and Table 1 show the hospitalization dates and positivity). Two out of these individuals died (Atl.15 and 16). To test whether additional monkeys were infected, fecal samples were collected from monkeys at the rescue center on two different dates (Fig. 1). All fecal samples collected from monkeys on 30–31 August (*n* = 12) tested positive for SARS-CoV-2, and five out of six monkey feces that were resampled after 20 days (20–21 September) were positive for SARS-CoV-2. One monkey (Atl.7), included during the first fecal sampling, was admitted at the veterinary hospital for a follow-up of previous hospitalization (non-COVID-19 related) and tested positive on September 21. Another monkey (Atl.3) that showed digestive signs plus lethargy and anorexia and was treated on site at the reserve also tested positive for SARS-CoV-2. Furthermore, on 6 December, Atl.10 monkey that had tested positive for SARS-CoV-2 on August was hospitalized for follow-up (non-COVID-19 related) and tested negative for SARS- CoV-2 (see sample details in Table S1).

Table 1 shows the SARS-CoV-2 positivity detected by RT-qPCR, the Cq value of RT-qPCR-positive and negative controls, clade assignment by Nextclade, and lineage assignment of the S gene sequence using hedgehog.

Despite performing different RNA extraction protocols and sequencing procedures, we were unable to recover complete viral genomes from all samples. However, we were able to sequence SARS-CoV-2 from 9 of the 16 monkeys (genomes that passed quality control were submitted to GenBank; accession numbers are detailed in Table S2), and for some individuals, we successfully sequenced the viral genome from both feces and nasal swabs (Table 1). Sample Alt.13 (swab) stands out as the only one to Nextclade’s quality control assessments, providing high confidence for its classification as belonging to the Omicron clade 22B and pango lineage BA.5.2. In contrast, the remaining samples [Atl.3, Atl.7 (swab), Atl.9, Atl.11, Atl.13 (feces and swab), Atl.14 (feces and swab), Atl.15, and Atl.16] exhibit numerous segments containing N’s. Nevertheless, Nextclade categorizes them as members of the Omicron Clade 22B and hedgehog (S-gene sequence) as Pango lineage B.1.1.529. Unfortunately, sequences belonging to Atl.1 and Atl.7 (feces) suffered from extensive missing data, leading to unsuccessful classification. However, in the case of sample Atl.1, the presence of amino acid substitutions, such as N: R203K and N: G204R, along with ORF1a: T3255I, hints at the involvement of SARS-CoV-2 in the broader 21M Omicron group (B.1.1.529). Similarly, mutations including N:P13L, N:R203K, N:G204R, ORF9b:P10S, S:Q954H, and S:N969K in Atl.11 suggest an association with the larger 21M Omicron (B.1.1.529) group. There are disparities in amino acid substitutions between the swab and fecal samples of the same individual; this is the case for Atl.13 and Atl.14; however, this is due to missing data.

Animal keeper’s results are also interesting as three out of four people (Ecu4266, Ecu4295, and Ecu 4297), who were actively involved in the deworming of the monkeys, tested positive for SARS-CoV-2 on 27 August. One of these individuals remained asymptomatic, while the other two experienced mild symptoms, primarily a sore throat, during the period spanning from 8 August to 14 August (as illustrated in Fig. 1; Table 1). Viral sequence analysis of samples obtained from these three keepers revealed the presence of extensive stretches of N’s. Furthermore, the sequences of samples Ecu4295 and Ecu4297 were classified within clade 22B (Omicron) and Pango lineage B.1.1.529, as elaborated in Table 1.

## DISCUSSION

The impact of the SARS-CoV-2 pandemic on wildlife has not been studied in detail, possibly because of the difficulty of detecting sick and infected animals in the wild. However, the large number of infected people worldwide, even in remote areas, makes it highly likely that unrecognized outbreaks have occurred in wildlife. This is especially true where people live near nature or domestic animals interact with wildlife. Unfortunately, surveillance of SARS-CoV-2 virus in animal species has been insufficient. Still, reports of outbreaks or clinical cases have helped us understand the impact of SARS-CoV-2 on animal health and the possible implications of an outbreak in the wild for persistence and new viral emergence. This is the case in our report, which describes the infection of SARS-CoV-2 in brown-headed spider monkeys (*Ateles fusciceps*) living in a rescue center specializing in the conservation of this species. It is interesting to note that most of the monkeys at the center are confined in artificial island enclosures with vegetation that simulate the monkeys’ natural habitat. In this environment, monkeys have no direct contact with humans, but indirect through food that is provided in baskets hoisted to the canopy by pulleys.

SARS-CoV-2 RNA was detected in all sampled monkeys (*n* = 16) at different time points. Initially, the manifestation of gastrointestinal symptoms like diarrhea, coupled with lethargy, fever, and anorexia in the first monkeys admitted to our care suggested that the recent deworming procedure, conducted a few days prior, might have distressed them. However, our team of veterinary experts, drawing from previous experiences (although unpublished) with another primate exhibiting similar symptoms, recognized the potential for SARS-CoV-2 infection. This suspicion was subsequently confirmed through SARS-CoV-2 positivity determined by RT-qPCR and validated via whole genome sequencing of the virus.

Indeed, sample Atl.13 (swab) passed all quality control assessments and was confidently classified into clade 22B (Omicron) and the Pango lineage BA.5.2. Similarly, samples obtained from monkeys (Atl.3, 7, 9, 11, 14, 15, and 16) and keepers (Ecu4295 and 4297) were categorized within clade 22B and lineage B.1.1.529. These samples exhibited lower quality due to extensive stretches of N’s, potentially affecting precise classification. Nonetheless, the presence of defining mutations such as M: D3N or N: P13L (https://covariants.org/variants/22B.Omicron), the heightened circulation of the Omicron variant at that time in Ecuador, and the successful classification of sample Atl.13 collectively led us to the conclusion that the SARS-CoV-2 clade 22B Omicron was indeed responsible for the infection.

Unfortunately, the substantial gaps in the genomic data prevent us from conducting phylogenetic analysis to elucidate potential transmission routes. However, it’s essential to emphasize that the sequences obtained do confirm the presence of the virus in the samples.

Our findings indicate that while deworming was not the direct cause of the clinical manifestation, it likely served as the vehicle for infection, given that the baits were handled by animal keepers who exhibited flu-like symptoms a week earlier. The scenario of viral spread from bait to deer was previously suggested (13), but not proven; however, it is well recognized that touching contaminated surfaces can be a source of infection (23). Another route of infection could have been through the handling of monkey’s food by animal keepers who were infected with SARS-CoV-2, and regrettably, they did so without wearing gloves or protective equipment. Additionally, SARS-CoV-2 has previously been reported to be shed in feces (24), and this shedding may have played a role in facilitating transmission within monkeys in the rescue center.

Unfortunately, we were unable to collect samples from baits, food, saimiri monkeys (which roam the surrounding area), or other animals in our SARS-CoV-2 analysis. Consequently, we cannot rule out these potential sources of infection. Nonetheless, direct transmission from humans to *A. fusciceps* at the site was implausible due to the absence of human-monkey contact.

Current reports of SARS-CoV-2 infection in wildlife are limited to the few individuals in which the virus has been detected. Signs of COVID-19 in animals have been reported to be highly variable (12), making it very difficult to predict how a wildlife population will respond to infection. Cases of SARS-CoV-2 infection in captive animals include pumas and lions in a zoo in South Africa (25), a snow leopard, a gorilla, an otter, a hyena, a fishing cat, a binturong, a coatimundi, and a lynx in the USA (26). There was also an outbreak in a gorilla group at the San Diego Zoo Safari Park; the eight gorillas recovered well and were further vaccinated (14, 16). In addition, wildlife reports in Brazil include deer, wild otters, and a case of a primate of the species *Mico melanurus* (13, 27, 28). Furthermore, non-human primates are well-studied models for the pathology and for evaluating vaccine candidates of SARS-CoV-2 (24, 29, 30). It has been shown that the immune response to SARS-CoV-2 is different between non-human primate species (30). Thus, it could be the reason that some individuals of *Ateles fusciceps* developed a strong response to the virus and others were asymptomatic.

Despite that this outbreak was not on a natural population of *A. fusciceps*, it affected 16 individuals. The impact of the infection caused 25% (4/16) hospitalization and 12.5% (2/16) mortality. Due to logistics problems and the distance from the rescue center, we did not receive enough tissues for histopathology, and the necropsy information was not taken. *Ateles fusciceps* is a critically endangered species, and it is estimated that less than 280 wildlife individuals remain in Ecuador (31, 32); furthermore, it is one of the 25 most endangered primates in the world (33). The information provided in our report on *A. fusciceps* contributes to understanding the impact that an outbreak of SARS-CoV-2 may have on this and other similar endangered monkey species. Nevertheless, it should be highlighted that the animals that recovered successfully received individualized medical care consisting of something that would not happen in the wild.

During the height of the SARS-CoV-2 pandemic, the virus reached the most remote areas of our planet at record speeds. The ability of the virus to infect a wide variety of animals makes it relevant to wildlife conservation and the One Health approach (34). The virus has shown to have a wide range of adaptation between mammal hosts, increasing the possibility of spillback events and the development of new variants of SARS-CoV-2 (13, 35). This emphasizes the need for regulations to protect wild animals from infected humans. As a result of this study, these regulations are being implemented in the rescue center and the risks of spillover or spillback are now being taken seriously. The use of protective equipment in wildlife hospitals, sanctuaries, or rescue centers should be mandatory when handling animals or their food, which will limit the transmission of infectious diseases (36). Another important aspect is to have regulations in national parks and ecological reserves that prevent human waste from encountering wild animals (37). It is important to establish channels of communication for this type of information to ensure that it is not distorted or misinterpreted and to avoid primates becoming the focus of reprisals (38). In this case, it was SARS-CoV-2; what will come next? In 2023, we are currently witnessing the tip of the iceberg of the impact of H5N1 influenza on birds and mammals around the world (39). Infectious disease surveillance in wildlife, zoos, markets, and even domestic animals should be conducted routinely, and results are publicly available. New genomic approaches make this feasible in many locations in the world. Only then will governments and world health organizations be able to make informed and timely decisions.

## Data Availability

SARS-CoV-2 genomes were submitted to GenBank under submissions: OQ630004:OQ630013[accn] and PP265987.

## SUPPLEMENTAL MATERIAL

Supplemental Material spectrum.02741-23-s0001.docx Tables S1 and Table S2 and Figure S1.

ASM does not own the copyrights to Supplemental Material that may be linked to, or accessed through, an article. The authors have granted ASM a non-exclusive, world-wide license to publish the Supplemental Material files. Please contact the corresponding author directly for reuse.
