# Supplementary material for: SARS-CoV-2 infection in brown-headed spider monkeys (Ateles fusciceps) at a wildlife rescue center on the coast of Ecuador—South America
Source: Microbiol Spectr. 2024 Feb 16;12(4):e02741-23. doi: 10.1128/spectrum.02741-23 (PMC10986564; doi:10.1128/spectrum.02741-23)
Supplement: Supplemental Material — Tables S1 and Table S2 and Figure S1. [file spectrum.02741-23-s0001.docx]

**Supplementary Material**

|  |  |  |  |  | Sample type | | | |
| --- | --- | --- | --- | --- | --- | --- | --- | --- |
| Sample | **Monkeys' habitat in refuge** | **Health status** | **Hospitalization date** | Deworming doses of nitazoxanide 100 mg/5ml* | **Oral swab** | **Feces first sampling** | **Feces second sampling** | **Tissue** |
| Atl.1 | Island 5 | Apparently healthy | - | 3,5 ml | 2022-09-17 | 2022-08-30 | 2022-09-21 | - |
| Atl.2 | Island 4 | Apparently healthy | - | 2,8 ml | - | 2022-08-30 | - | - |
| Atl.3 | Island 5 | Treated in situ | - | 0,9 ml | 2022-09-17 | 2022-08-30 | 2022-09-21 | - |
| Atl.4 | Enclosure 4 | Apparently healthy | - | 2,8 ml | - | 2022-08-30 | - | - |
| Atl.5 | Island 5 | Apparently healthy | - | 2,8 ml | - | 2022-08-30 | 2022-09-21 | - |
| Atl.6 | Island 5 | Apparently healthy | - | 0,9 ml | - | 2022-08-30 | 2022-09-21 | - |
| Atl.7 | Enclosure 1 | Hospitalized | 2022-09-21 | 3,5 ml | 2022-09-21** | 2022-08-30 | - | - |
| Atl.8 | Enclosure 4 | Apparently healthy | - | 2,8 ml | - | 2022-08-31 | - | - |
| Atl.9 | Island 6 | Apparently healthy | - | 1,6 ml | - | 2022-08-31 | - | - |
| Atl.10 | Enclosure 3 | Apparently healthy | - | 3,5 ml | - | 2022-08-31 | 2022-12-06** | - |
| Atl.11 | Island 5 | Apparently healthy | - | *** | - | 2022-08-31 | 2022-09-20 | - |
| Atl.12 | Island 6 | Apparently healthy | - | 2,8 ml | - | 2022-08-31 | 2022-09-21 | - |
| Atl.13 | Island 6 | Hospitalized | 2022-08-25 | 2,8 ml | 2022-08-25 | 2022-08-20 | - | - |
| Atl.14 | Island 6 | Hospitalized | 2022-08-27 | *** | 2022-08-29 | 2022-09-20 | - | - |
| Atl.15 | Island 5 | Hospitalized | 2022-08-23 | 2,8 ml | 2022-08-24 | - | - | - |
| Atl.16 | Enclosure 2 | Dead before hospital arrival | 2022-08-23 | 2,8 ml | - | - | - | 2022-08-24 |

**Table S1.**  Collection dates, sampling sites, and health status of the monkeys.

*Visual approximation of animal weight used to calculate deworming doses

**Collected when hospitalized for non-COVID-19 reasons

*** We were not able to deworm this monkey.

Table S1 shows the location of the monkeys inside the wildlife rescue center, whether they were hospitalized, and the different sampling dates.

**Table S2.** Clade, lineage assignment, and AA substitutions of sequenced samples from monkeys and animal keepers.

| Sample | Type | GenBank accession | Missing (N´s) | Clade | Lineage | AA Substitutions |
| --- | --- | --- | --- | --- | --- | --- |
| **Monkeys** |  |  |  |  |  |  |
| Atl.1 | Swab | Not submitted | 24837* * | Unassigned | Unassigned | N:R203K,N:G204R,N:Q349K,N:S413R,ORF1a:T3090I,ORF1a:T3255I,ORF1b:P314L |
|  | Feces | Not submitted | 27831 * * | Unassigned | Unassigned | ORF1a:I3758V |
| Atl.3 | Swab | OQ630011 | 9031 * * | 22B (Omicron) | Unassigned | E:T9I,M:Q19E,M:A63T,N:P13L,N:R203K,N:G204R,N:S413R,ORF1a:L3027F,ORF1a:T3090I,ORF1a:T3255I,ORF1a:P3395H,ORF1b:P314L,ORF1b:I1566V,ORF1b:T2163I,ORF3a:T223I,ORF9b:P10S,S:T19I,S:A27S,S:G142D,S:V213G,S:D614G,S:H655Y,S:N679K,S:P681H,S:Q954H,S:N969K |
|  | Feces | OQ630010 | 9021 * * | 22B (Omicron) | B.1.1.529 | M:D3N,M:Q19E,M:A63T,N:P13L,N:R203K,N:G204R,N:S413R,ORF1a:S135R,ORF1a:G1307S,ORF1a:I2377V,ORF1a:L3027F,ORF1a:T3090I,ORF1a:T3255I,ORF1b:P314L,ORF9b:P10S,S:T19I,S:A27S,S:G142D,S:V213G,S:G339D,S:S371F,S:S373P,S:S375F,S:T376A,S:D405N,S:R408S,S:K417N,S:D614G,S:H655Y,S:N679K,S:P681H,S:N764K,S:D796Y,S:Q954H,S:N969K |
| Atl.7 | Swab | Not submitted | 20654 * * | 22B (Omicron) | Unassigned | M:A63T,N:P13L,ORF1a:S135R,ORF1a:T3090I,ORF3a:T223I,ORF9b:P10S,S:G339D,S:D405N,S:R408S,S:K417N,S:Q954H,S:N969K |
|  | Feces | Not submitted | 26159 * * | Unassigned | Unassigned | N:P13L,N:R203K,N:G204R,ORF9b:P10S,S:Q954H,S:N969K |
| Atl.9 | Feces | PP265987* | 16070 * * | 22B (Omicron) | B.1.1.529 | M:A63T,N:P13L,N:R203K,N:G204R,N:S413R,ORF1a:G1307S,ORF1a:L3027F,ORF1a:T3090I,ORF1a:T3255I,ORF1a:P3395H,ORF1b:P314L,ORF1b:T2163I,ORF3a:T223I,ORF9b:P10S,S:T19I,S:A27S,S:G142D,S:V213G,S:S297*,S:Q954H,S:N969K |
| Atl.11 | Feces | OQ630004 | 5071 * * | 22B (Omicron) | B.1.1.529 | M:D3N,M:Q19E,M:A63T,N:P13L,N:R203K,N:G204R,ORF1a:G1307S,ORF1a:L3027F,ORF1a:T3090I,ORF1a:T3255I,ORF1a:P3395H,ORF1b:P314L,ORF1b:T2163I,ORF9b:P10S,S:T19I,S:A27S,S:G142D,S:V213G,S:G339D,S:S371F,S:S373P,S:S375F,S:T376A,S:D405N,S:R408S,S:K417N,S:D614G,S:H655Y,S:N679K,S:P681H,S:N764K,S:D796Y,S:Q954H,S:N969K |
| Atl.13 | Swab | OQ630005 | 1033 | 22B (Omicron) | BA.5.2 | E:T9I,M:D3N,M:Q19E,M:A63T,N:P13L,N:R203K,N:G204R,N:S413R,ORF1a:S135R,ORF1a:T842I,ORF1a:G1307S,ORF1a:L3027F,ORF1a:T3090I,ORF1a:T3255I,ORF1a:P3395H,ORF1b:P314L,ORF1b:T1050N,ORF1b:R1315C,ORF1b:T2163I,ORF3a:T223I,ORF9b:P10S,ORF9b:D16G,S:T19I,S:A27S,S:G142D,S:V213G,S:G339D,S:R346S,S:S371F,S:S373P,S:S375F,S:T376A,S:D405N,S:K417N,S:D614G,S:H655Y,S:N679K,S:P681H,S:N764K,S:D796Y,S:Q954H,S:N969K |
|  | Feces | Not submitted | 26455 * * | 22B (Omicron) | Unassigned | N:P13L,N:R203K,N:G204R,ORF1a:P3395H,ORF1b:P314L,ORF9b:P10S |
| Atl.14 | Swab | OQ630007 | 6565 * * | 22B (Omicron) | B.1.1.529 | E:T9I,M:Q19E,M:A63T,N:P13L,N:R203K,N:G204R,N:S413R,ORF1a:S135R,ORF1a:G1307S,ORF1a:T3090I,ORF1a:T3255I,ORF1a:P3395H,ORF1b:P314L,ORF1b:T2163I,ORF3a:T223I,ORF9b:P10S,S:T19I,S:A27S,S:G142D,S:V213G,S:D614G,S:H655Y,S:N679K,S:P681H,S:N764K,S:D796Y,S:Q954H,S:N969K |
|  | Feces | OQ630006 | 4743 * * | 22B (Omicron) | B.1.1.529 | E:T9I,M:D3N,M:Q19E,M:A63T,N:P13L,N:R203K,N:G204R,N:S413R,ORF1a:T842I,ORF1a:G1307S,ORF1a:L3027F,ORF1a:T3090I,ORF1a:T3255I,ORF1a:P3395H,ORF1b:P314L,ORF1b:R1315C,ORF1b:I1566V,ORF1b:P1666T,ORF1b:T2163I,ORF3a:T223I,ORF9b:P10F,S:T19I,S:A27S,S:G142D,S:V213G,S:G339D,S:S371F,S:S373P,S:S375F,S:T376A,S:D405N,S:R408S,S:K417N,S:D614G,S:H655Y,S:N679K,S:P681H,S:N764K,S:D796Y,S:Q954H,S:N969K |
| Atl.15 | Swab | OQ630008 | 3351 * * | 22B (Omicron) | B.1.1.529 | E:T9I,M:D3N,M:Q19E,M:A63T,N:P13L,N:S413R,ORF1a:S135R,ORF1a:T842I,ORF1a:G1307S,ORF1a:L3027F,ORF1a:T3090I,ORF1a:T3255I,ORF1a:P3395H,ORF1b:P314L,ORF1b:T2163I,ORF3a:T223I,ORF9b:P10S,S:T19I,S:A27S,S:G142D,S:V213G,S:D614G,S:H655Y,S:N679K,S:P681H,S:N764K,S:D796Y,S:Q954H,S:N969K |
| Atl.16 | Tissue | OQ630009 | 10507 * * | 22B (Omicron) | B.1.1.529 | N:E31-,N:R32-,N:S33-,ORF9b:E27-,ORF9b:N28-,ORF9b:A29- |
| **Monkey keepers** |  |  |  |  |  |  |
| Ecu4266 | Swab | Not submitted | 20358 * * | 22B (Omicron) | Unassigned | N:P13L,N:R203K,N:G204R,N:S413R,ORF1a:S135R,ORF1a:T3090I,ORF1b:T2163I,ORF9b:P10S,S:Q954H,S:N969K |
| Ecu4295 | Swab | OQ630012 | 7691 * * | 22B (Omicron) | B.1.1.529 | E:T9I,M:D3N,M:Q19E,M:A63T,N:P13L,N:R203K,N:G204R,N:S413R,ORF1a:S135R,ORF1a:G1307S,ORF1a:L3027F,ORF1a:T3090I,ORF1a:T3255I,ORF1a:P3395H,ORF1b:P314L,ORF1b:I1566V,ORF1b:T2163I,ORF3a:T223I,ORF9b:P10S,S:T19I,S:A27S,S:G142D,S:V213G,S:G339D,S:S371F,S:S373P,S:S375F,S:T376A,S:D405N,S:R408S,S:K417N,S:D614G,S:H655Y,S:N679K,S:P681H,S:Q954H,S:N969K |
| Ecu4297 | Swab | OQ630013 | 6736 * * | 22B (Omicron) | B.1.1.529 | E:T9I,M:D3N,M:Q19E,M:A63T,N:P13L,N:R203K,N:G204R,N:S413R,ORF1a:S135R,ORF1a:G1307S,ORF1a:L3027F,ORF1a:T3090I,ORF1a:T3255I,ORF1a:P3395H,ORF1b:P314L,ORF1b:T2163I,ORF3a:T223I,ORF9b:P10S,ORF9b:D16G,S:T19I,S:A27S,S:G142D,S:V213G,S:D614G,S:H655Y,S:N679K,S:P681H,S:N764K,S:D796Y,S:Q954H,S:N969K |

* Partial S gene sequence submitted

* * Low quality due to long stretches of N’s

Appendix Table 2 shows the clade assignment with Nextclade along with the amino acid substitutions compared to the Wuhan-Hu-1/2019 reference genome and missing data of the viral genome (N’s), and the lineage assignment of S-gene sequences with more than 50% of coverage with the software hedgehog. Sequences that passed quality controls were submitted to gene GenBank.


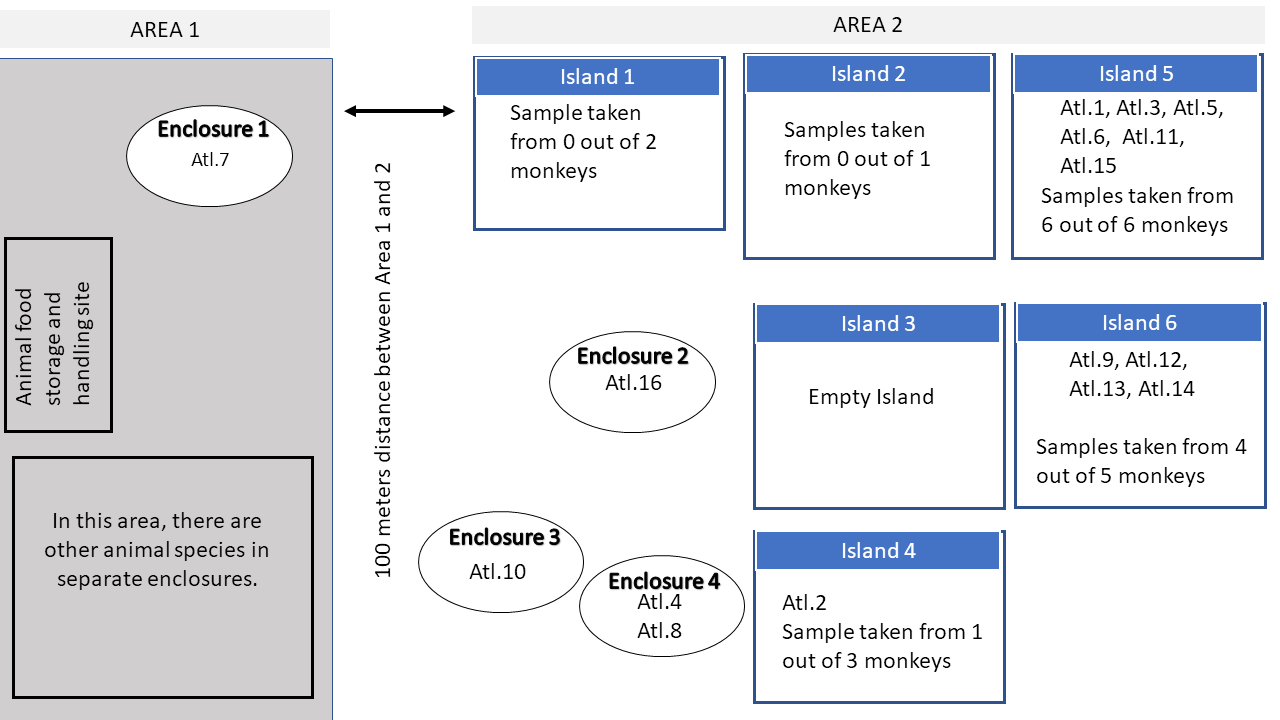


**Figure S1.** Schematic layout of the place where brown-headed spider monkeys, *Ateles fusciceps* (Atl.), are kept inside the rescue center. Code for each sampled monkey is detailed for each island or enclosure.
